# Polymer-Encased Nanodiscs and Polymer Nanodiscs: New Platforms for Membrane Protein Research and Applications

**DOI:** 10.3389/fbioe.2020.598450

**Published:** 2020-11-16

**Authors:** Angela Chen, Elleana J. Majdinasab, Mariana C. Fiori, Hongjun Liang, Guillermo A. Altenberg

**Affiliations:** ^1^School of Medicine, Texas Tech University Health Sciences Center, Lubbock, TX, United States; ^2^Department of Cell Physiology and Molecular Biophysics, and Center for Membrane Protein Research, School of Medicine, Texas Tech University Health Sciences Center, Lubbock, TX, United States

**Keywords:** copolymer, lipid, Lipodisq, membrane-mimetic, method, native nanodisc, styrene maleic acid, synthetic membrane

## Abstract

Membrane proteins (MPs) are essential to many organisms’ major functions. They are notorious for being difficult to isolate and study, and mimicking native conditions for studies *in vitro* has proved to be a challenge. Lipid nanodiscs are among the most promising platforms for MP reconstitution, but they contain a relatively labile lipid bilayer and their use requires previous protein solubilization in detergent. These limitations have led to the testing of copolymers in new types of nanodisc platforms. Polymer-encased nanodiscs and polymer nanodiscs support functional MPs and address some of the limitations present in other MP reconstitution platforms. In this review, we provide a summary of recent developments in the use of polymers in nanodiscs.

## Introduction

MPs are essential for cell homeostasis, signaling, transport, and energy production. They play critical roles in normal function and human disease, and as such they are important drug targets (Overington et al., 2006). MPs have an integral role in the pathophysiology of diseases, such as cystic fibrosis, hypertension, diabetes, cerebrovascular accidents, cardiac infarcts, and neurodegenerative diseases, to name a few (Lee and White, 2009; Orellana et al., 2014; Beyder and Farrugia, 2016; Kumar et al., 2016; Schmidt et al., 2016; Zaucha et al., 2020). At least 50% of current druggable targets are classified as MPs, and about half of all known small molecule drugs bind to them (Russell and Eggleston, 2000; Overington et al., 2006). Therefore, there is high priority in understanding MPs because of their importance to improve therapeutics for many medical conditions. Yet, progress in the MP field has been slow in comparison to that of other biomolecules because of difficulties in overexpression, membrane extraction, and purification, which frequently vary even for proteins within the same family (Seddon et al., 2004; Hardy et al., 2018). Maintaining an environment conducive to MP function and stability for evaluation *in vitro* is still a major challenge.

Studies of proteins in their native environment are critical to understanding their function and regulation in organisms and cells, but details on their structures and mechanisms at the atomic and molecular level are better investigated on purified proteins under well-controlled conditions. In the case of MPs, a complete understanding requires working with purified proteins reconstituted in model membranes that are robust enough to support research in an abiotic environment for extended periods of time. And yet, MPs are notoriously difficult to isolate and study due to the hurdle of discovering native-membrane-mimicking environments that cater to their stability while also support their functions. Ideally, a purified MP of interest should be incorporated in a membrane in which it can operate as it would in the native cell membrane, but in a solubilized and stable state where it can be subjected to analyses of its structure, function, and regulation.

**FIGURE 1:**
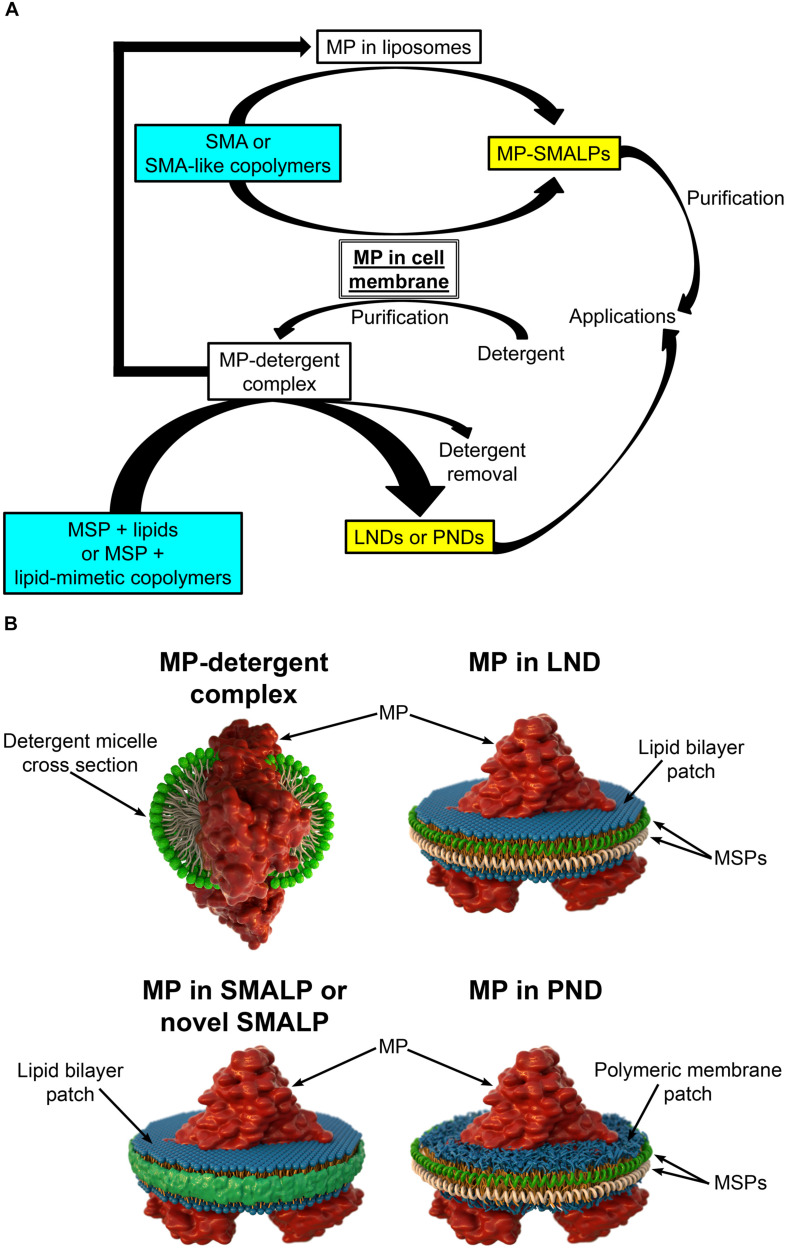
Different types of nanodiscs. **(A)** Schematic representation of the steps involved in the production of different types of membrane protein (MP)-loaded nanodiscs. Note that styrene maleic acid (SMA) and SMA-like copolymers can yield SMA lipid particles (SMALPs or novel SMALPs) in a single solubilization/reconstitution step without the need for detergents if cell membranes are the starting material. The soluble MPs reconstituted in SMALPs can then be purified in the absence of detergent. In contrast, production of lipid (LNDs) and polymer (PNDs) nanodiscs requires MPs solubilized in detergent. **(B)** Schematic representation of a MP in a detergent micelle and in different types of nanodiscs.

In most cases, studies of purified MPs include expression through recombinant methods followed by extraction from membranes using detergents to produce the proteins solubilized in detergent micelles (Figure 1; Seddon et al., 2004; Hardy et al., 2018; Stroud et al., 2018). Once solubilized, purification can proceed using methodologies typically employed to purify soluble proteins, but in the continuous presence of detergent to keep the MP in a soluble state. The purification methodologies frequently include liquid chromatography based on fusion tags added to the protein (e.g., His tag, FLAG tag) and/or on the intrinsic properties of the protein (e.g., size-exclusion chromatography, ion-exchange chromatography) (Seddon et al., 2004; Morrison et al., 2016). While detergents are the most common agents used for MP extraction, finding the best detergent can be time consuming (Le Maire et al., 2000). Even if the best detergent is found, decreased MP stability and alterations in MP structure and function are possible (Leitz et al., 2006; Brewer et al., 2011; Hagn et al., 2013; Denisov and Sligar, 2016; Viegas et al., 2016; Zoghbi et al., 2016). Therefore, it is desirable to reconstitute the MPs into a lipid-bilayer-like environment. The simplest solution is adding lipids to the detergent to form detergent-lipid mixed micelles (Seddon et al., 2004; Skrzypek et al., 2018). However, heterogeneities make these micelles suboptimal for biophysical studies (Zoghbi et al., 2017). Drawbacks on traditional methods have prompted investigations of new methodologies for the extraction, isolation, and reconstitution of MPs that are aimed at overcoming logistical hurdles. There are several platforms available for MP reconstitution, including planar lipid bilayers, liposomes, and nanodiscs (Skrzypek et al., 2018). Here we focus on the latter, which constitute an MP platform of excellent properties that offers great potential.

## Lipid Nanodiscs

Lipid nanodiscs (LNDs) are composed of a disc-shaped phospholipid bilayer surrounded by a two-molecule belt of a membrane scaffold protein (MSP) (Figure 1B), a protein derived from apolipoprotein A1, a major component of serum high-density lipoprotein complexes (Denisov and Sligar, 2016). Usually, these nanostructures are referred to as “nanodiscs.” However, here we use the term LNDs to differentiate them from other types of nanodiscs discussed below. LNDs contain a lipid bilayer that provides MPs with stability and access from both sides of the membrane. MP-loaded LNDs are nanometer-scale structures that can be produced in a monodisperse solution and are therefore suitable for most biochemical and biophysical assays, including optical and NMR spectroscopies, as well as cryo-electron microscopy (Denisov and Sligar, 2016, 2017; Thonghin et al., 2018). Although LNDs have emerged as an excellent MP platform for many applications, reconstitution into LNDs requires the use of detergents for extraction of the MP from the membrane (Figure 1A). Thus, composition of the lipid bilayer in the nanodiscs is not the same as that of the native lipid bilayer but instead that of the composition selected during LNDs preparation, potentially affecting protein structure, activity, and regulation. Furthermore, even though LNDs are still not broadly used in biotechnology and biomedical applications, the labile nature of lipid bilayers and poor chemical versatility could restrict those applications. Due to these limitations, there is a continuous interest in new developments, which has resulted in new types of nanodiscs based on the use of polymers. These can have advantages over LNDs for some applications and can replace detergents for the extraction of MPs from membranes and for protein purification.

## SMALPs

Nanoparticles based on styrene maleic acid (SMA) copolymers constitute a promising approach for MP extraction and purification in a native bilayer environment (Dorr et al., 2016; Lee et al., 2016; Stroud et al., 2018). Solubilization of membranes with SMAs results in the formation of SMA lipid particles (SMALPs), nanostructures consisting of the native lipid bilayer encircled by a belt of amphipathic SMA copolymers that replace the MSPs of LNDs (Figure 1; Morrison et al., 2016; Stroud et al., 2018). SMALPs are also referred to as native nanodiscs or Lipodisqs. SMA copolymers with various styrene-to-maleic acid ratios can be synthesized to form polymers with different hydrophobicity and solubilization properties (Morrison et al., 2016). Direct solubilization of membranes by SMAs allows for purification of MPs in the absence of detergents (Figure 1A), avoiding their detrimental effects (Stroud et al., 2018). Because MP-loaded SMALPs consist of a small patch of the native lipid bilayer with a MP, stability of the latter is expected to be higher than that of the protein solubilized in detergent, allowing for studies of structure and function in a native-like environment (Dorr et al., 2014; Lee et al., 2016; Parmar et al., 2018; Stroud et al., 2018; Routledge et al., 2020). Although transport measurements cannot be performed in SMALPs (or LNDs), both sides of the MP are accessible for straightforward measurements of activity and structural changes that result from binding of ligands and modulators.

While SMAs are promising alternatives to traditional solubilization and reconstitution methods, their use presents serious limitations. First, there is a limited pH range in which SMAs are stable and will form SMALPs, with the styrene-to-maleic acid ratio and the charge of carboxylic groups on the maleic acid units affecting the pH range in which SMALPs are soluble (Banerjee et al., 2012; Fiori et al., 2017a; Ravula et al., 2017a; Hall et al., 2018a). At lower pHs, SMAs become insoluble because of their increased hydrophobicity, making them unsuitable for forming SMALPs with MPs that function at low pH or for downstream applications that involve studies at pH <∼6.5 (Fiori et al., 2017a; Ravula et al., 2017a, 2019). Second, the size of SMALPs may be too small to contain “large” MPs. The average diameter of SMALPs is 6–9 nm, which can restrict the proteins that can be studied (Fiori et al., 2017a, 2020; Ravula et al., 2017b, 2019). Third, SMA polymers can bind divalent cations, such as Mg^2+^ and Ca^2+^, reducing the charge of the maleic acid units, which results in precipitation of the copolymers out of solution (Fiori et al., 2017a, 2020; Ravula et al., 2017a). The sensitivity to divalent cations can be restrictive to downstream analyses that require their presence, such as measurements of ATPase activities (Fiori et al., 2017a).

Recently, a method was developed to improve downstream applications of MPs solubilized with SMA (Hesketh et al., 2020). The approach takes advantage of the precipitation of the SMA with Mg^2+^ while maintaining the MP soluble with added amphipols or detergents (Hesketh et al., 2020). This platform exchange method results in a product that is compatible with millimolar concentrations of Mg^2+^, displays increased homogeneity when compared to SMALPs, and is suitable for mass spectrometry, and probably other downstream applications, such as electron microscopy (Hesketh et al., 2020). Since native membrane lipids present in the SMALPs are transferred to the new exchange platform (Hesketh et al., 2020), stability of the purified MP may be enhanced and studies on the regulation of function by native lipids are possible.

## Novel SMALPs

Given the difficulties in using traditional SMAs, there has been emerging research aimed at creating new SMA-like copolymers with broader buffer compatibilities that can also form SMALPs of larger sizes. The maleic acid groups of SMA are easily accessible and amenable to modification, enabling the addition of different chemical groups to create new polymers. One of the promising SMA-like copolymers that we developed to address the buffer incompatibilities of SMAs and the size limitations of SMALPs are the zwitterionic styrene maleic amides (zSMAs) (Fiori et al., 2017a, 2020). In these copolymers, the anionic carboxyl acids are replaced with zwitterionic phosphatidylcholine groups. Contrary to SMAs, zSMAs do not precipitate out of solution at low pH or in the presence of polycations (Fiori et al., 2017a). Moreover, well-defined zSMAs of varying copolymer sizes can be used to control the diameter of the resulting zSMALPs (Fiori et al., 2017a). There are other new copolymers that have been produced recently which are also stable at acidic pH, do not precipitate in the presence of millimolar concentrations of divalent cations, and/or form nanostructures larger than SMALPs. These include diisobutylene/maleic acid copolymer (DIBMA), poly(styrene-co-maleimide) (SMI), SMA-ethanolamine (SMA-EA), SMA-ethylenediamine (SMA-ED; a zwitterionic form of SMA-EA), SMAd-A (formed by dehydrating SMA-ED), styrene maleimide quaternary ammonium (SMA-QA; a positively charged SMA derivative), and SMA-SH (with thiol groups on the polar side chains) (Lindhoud et al., 2016; Oluwole et al., 2017; Ravula et al., 2017a,b, 2019; Hall et al., 2018a). The latter is unique in that fluorescent tags and dyes can be attached to it, permitting SMALP affinity purification and fluorescence detection among many potential applications (Lindhoud et al., 2016). For zSMA and other SMA-like copolymers synthesized by reversible addition-fragmentation chain transfer, or RAFT polymerization, it is simple and convenient to convert the chain-transfer moiety on the polymers to a thiol group for affinity purification or fluorescence detection.

As SMAs, SMA-like copolymers can be used to extract and reconstitute MPs directly from cell membranes in a single-step (Figure 1A; Lee et al., 2016; Stroud et al., 2018). This creates a clear advantage over LNDs in that detergents are not necessary, and the composition of the lipid bilayer that surrounds the reconstituted MP is expected to be less drastically different from that in the native membranes (Lee et al., 2016; Stroud et al., 2018). The latter is important when studies are aimed to reproduce native-like conditions as closely as possible. In addition, purified MPs reconstituted in liposomes can be re-solubilized to form traditional or novel SMALPs where the lipid composition of the bilayer can be controlled through the composition of the liposomes (Figure 1A; Ravula et al., 2017b; Fiori et al., 2020).

In the case of zSMAs, our copolymers series was produced by RAFT polymerization (Fiori et al., 2017a, 2020). Compared to those produced by other methods, RAFT polymerization yields polymers of more uniform size (lower polydispersity index), which seems to be an advantage for solubilization/reconstitution (Smith et al., 2017; Fiori et al., 2020). Although a gradient increase of styrene repeating units at the end of individual copolymer molecules is possible during any polymerization process, it can be minimized in RAFT polymerization by precisely controlling the conversion of styrene in the styrene-maleic anhydride random copolymers (Fiori et al., 2017a, 2020; Smith et al., 2017). This is important because a terminal polystyrene homopolymer segment could reduce solubilization efficiency (Hall et al., 2018b).

Using RAFT polymerization, we produced zSMA copolymers of varying sizes and styrene-to-maleic amide molar ratios (Fiori et al., 2020). Testing of these copolymers showed that they are effective agents to solubilize different MPs directly from membranes or from proteoliposomes containing purified MPs (Fiori et al., 2017a, 2020). The resulting nanoparticles have hydrodynamic diameters of 10–30 nm, depending on the polymer chain size (Fiori et al., 2017a). Extensive studies of solubilization into zSMALPs under varied experimental conditions indicate that the solubilization/reconstitution by zSMAs is better than that by the equivalent SMAs, and is very robust in the sense that it occurs with similar efficiency under a variety of conditions that include differences in buffer composition, pH and temperature (Fiori et al., 2020). This has the important practical implication that establishing solubilization/reconstitution protocols will not necessitate extensive trials.

Unexpectedly, we found that a zSMA with a 2:1 styrene:maleic amide molar ratio was better than a zSMA with a 1:1 ratio at thermal stabilization of the ATP-binding cassette protein MsbA, but the ATPase activity of MsbA was higher with the 1:1 zSMA (Fiori et al., 2020). Although the underlying reasons for the differences are unclear, the results point to our limited understanding of the interactions of the SMA-like copolymers with the lipid bilayer and/or the reconstituted MPs. Regardless, we can appreciate the objective advantages of the new SMA-like copolymers in the context of studies at low pH or in the presence of divalent cations, as well as the greater control on nanodisc size and stability.

## Polymer Nanodiscs (PNDs)

As mentioned above, even though the use of LNDs and SMALP-like nanoparticles in biotechnology and biomedical applications is so far very limited, it is obvious that the fluid and labile characteristics of the lipid bilayer in both types of nanodiscs will constitute a limitation for stability and long-term storage without disturbances in the nanodiscs structure. Although studies of nanodiscs stability are very scarce, we found that LNDs start to aggregate within a week, even at 4°C, and that they aggregate much faster at higher temperatures or after freeze/thaw cycles (Fiori et al., 2017b).

Synthetic polymeric membranes seek to retain the soft properties of the native bilayers combined with the stability and chemical flexibility of polymers. Because natural membranes are intricate and complex, the custom design and synthesis of polymers allow for more control and versatility of the membrane properties and to tailor these properties to specific research, biotechnology, and biomedical applications. Like natural lipid bilayers, synthetic membranes can incorporate MPs as well as hydrophobic and amphipathic compounds (Goers et al., 2018; Yorulmaz Avsar et al., 2019). Given that lipids can have specific effects on MPs (Alvis et al., 2003; Opekarova and Tanner, 2003), it is notable that endogenous lipids can be inserted, if necessary, and function normally within these synthetic membranes (Seneviratne et al., 2018).

MPs, such as aquaporin and ATP synthase have been incorporated into polydimethylsiloxane (PDMS)-based triblock copolymer membranes, which are viscous and fluid at room temperature (Meier et al., 2000; Kumar et al., 2007; Kowal et al., 2014; Itel et al., 2015). To address the lack of stability of these fluid membranes, we have developed polybutadiene (PBD)- and polystyrene (PS)-based block copolymers, both of which support successful functional reconstitution of MPs while displaying remarkable versatility and stability (Hua et al., 2011; Kuang et al., 2014a,b; Fiori et al., 2017b). Thus, through varying the composition and structure of the block copolymers to change flexibility and fluidity of the self-assembled polymeric membranes, synthetic amphiphilic block copolymers are emerging as new tools for the reconstitution of MPs with preservation of their structural integrity and function.

Synthetic polymeric membranes have been used in various platform types for reconstitution of MPs, including polymer nanodiscs (PNDs) (Meier et al., 2000; Kumar et al., 2007; Hua et al., 2011; Kuang et al., 2014b; Kowal et al., 2015; Fiori et al., 2017b). PNDs constitute a new platform developed to address the limitation in stability and versatility of LNDs and SMALPs. PNDs do not contain biological lipids, but the membrane patch rather consists entirely of synthetic amphiphilic block copolymers (Figure 1). The only PNDs reported so far are our discoidal amphiphilic block polymer membrane patches encased within MSPs (Fiori et al., 2017b). In these PNDs the hydrophobic core of the lipid bilayer was replaced with hydrogenated polybutadiene, and the hydrophilic lipid headgroup moieties were replaced with the positively charged poly vinyl methylpyridine. These PNDs are easily produced and, as LNDs, assemble spontaneously under the appropriate conditions, forming uniform nanodiscs similar to LNDs in size when the same type of MSP was used (Fiori et al., 2017b). The ATPase activity of the ATP-binding cassette pump MsbA reconstituted in these PNDs was several folds higher than that of the MP solubilized in detergents, and equivalent to that of MsbA in LNDs (Fiori et al., 2017b). The PNDs showed much improved stability *vis-à-vis* LNDs, including lack of aggregation when stored at temperatures between 4 and 37°C, and following a freeze/thaw cycle (Fiori et al., 2017b). These studies indicate that PNDs can support functional MPs and showcase their remarkable stability.

## Availability of Nanodisc Components

Since some of the different types of MP platforms described in this review are fairly new, in this section we give information about the availability of the nanodisc components. The information presented is not exhaustive, but aimed at providing a starting point for interested potential users since new copolymers are becoming available rapidly, traditional components, such as MSPs and lipids are available from several sources, and new copolymers are generally available from their developers before they are commercially available. A large number of detergents and phospholipids, as well as cholesterol and other lipids, are available from Avanti Polar Lipids^1^, Millipore Sigma^2^, Larodan^3^, Anatrace^4^, and Cube Biotech^5^, among many sources. Purified MSPs are available from sources, such as Millipore Sigma and Cube Biotech, and plasmids for the expression and purification of MSPs in-house are available from Addgene^6^. SMAs and some SMA-like copolymers are available from Millipore Sigma, Orbiscope^7^, Anatrace, Avanti Polar Lipids, Cube Biotech, and Polyscope^8^. Lipid mimetic copolymers are not commercially available yet, but as with other new copolymers not yet in the market, they can be obtained on limited bases from the laboratories that developed them. The SMALP Network^9^ is a good source to learn about new copolymers as they become available.

## Conclusion, Future Developments, and Perspectives

The use of copolymers is a promising addition to MP research and applications. Two major advantages of copolymers over biomolecules, such as MSPs and lipids are their stability and amenability to engineering modifications tailored to specific applications. New developments in amphiphilic copolymers, such as SMA-like polymers offer the promise of direct, simultaneous solubilization of MPs in the absence of detergent, and reconstitution in the native membrane lipids (Ravula et al., 2019). Contrary to SMAs, they enjoy size control of the resulting SMALPs and compatibility with a variety of buffers (Fiori et al., 2017a; Ravula et al., 2019). The development of new SMA-like polymers with improved properties has accelerated during the last few years, but testing of the copolymers and characterization of the resulting nanostructures still lag behind.

Whereas, LNDs tend to aggregate under conditions, such as physiological mammalian temperature needed for many experiments as well as freeze/thaw cycles experienced during transport/storage/use, PNDs do not, rendering them conducive to functioning in abiotic environments for long periods of time under broader experimental conditions (Fiori et al., 2017b). PNDs’ high chemical versatility and membrane stability may potentially lead to exciting developments. It is interesting that a variety of MPs can be functionally reconstituted in synthetic polymeric membranes, including the water channel aquaporin, potassium channels, the light-driven proton pump proteorhodopsin, a bacterial reaction center, an ATP synthase, the G-protein coupled receptor protein rhodopsin, and the ATP-binding cassette transporter MsbA (Kumar et al., 2007; Hua et al., 2011; Kowal et al., 2014; Kuang et al., 2014b; Itel et al., 2015; Fiori et al., 2017b). Even more remarkably, proteorhodopsin displays a “normal” photocycle at the structural and functional level when reconstituted in “frozen” polysterene-based membranes of glass transition temperatures >70°C, which display superior bulk-state stability when compared to other synthetic membranes (Kuang et al., 2014a,b).

A representation and a comparative summary of the different types of nanodiscs described here are presented in Figure 1B and Table 1, respectively. Despite the recent developments, more studies on the basic characterization of the new copolymers and their applications are needed. In this context, the production of PNDs with highly stable polysterene-based membranes, and the possibility of engineering fully polymeric nanodiscs combining new SMA-like copolymers and synthetic polymeric membranes seem promising avenues to explore.

**TABLE 1:** Comparison of different types of nanodiscs.

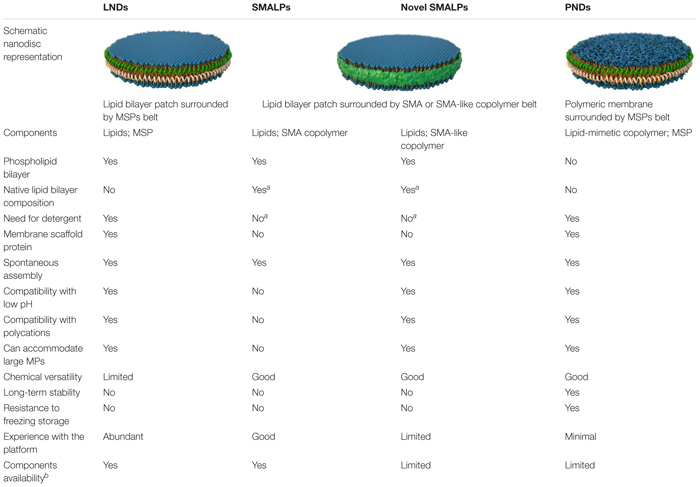

*^*a*^When solubilization/reconstitution starts from membranes, but detergent is needed if proteoliposomes are the starting material to produce SMALPs or novel SMALPs (see Figure 1). ^*b*^See section “Availability of Nanodisc Components” for details.*

## Author Contributions

AC, EM, MF, HL, and GA wrote the manuscript. All authors approved the final manuscript.

## Conflict of Interest

The authors declare that the research was conducted in the absence of any commercial or financial relationships that could be construed as a potential conflict of interest.

## Footnotes

1 https://avantilipids.com/

2 https://www.emdmillipore.com/

3 https://www.larodan.com/

4 https://www.anatrace.com/

5 https://cube-biotech.com/

6 https://www.addgene.org/

7 https://www.orbiscope.com/

8 https://polyscope.eu/

9 http://www.smalp.net/
